# Autistic- and attention-deficit/hyperactivity disorder-like traits: differential associations with burnout, depression and anxiety, and empathy among Japanese junior residents

**DOI:** 10.3389/fpsyt.2026.1858994

**Published:** 2026-07-09

**Authors:** Takafumi Watanabe, Toshiya Ishii, Tatsuo Akechi

**Affiliations:** 1 Department of Psychiatry, Nagoya City University Graduate School of Medical Sciences, Nagoya, Japan; 2 Gifu Hospital, Gifu, Japan

**Keywords:** burnout, depression, empathy, neurodevelopmental traits, physicians, psychological flexibility

## Abstract

**Introduction:**

Burnout, depression, and anxiety are major concerns among physicians because they affect individual well-being, patient care, and healthcare systems. Neurodevelopmental traits, including autistic-like traits (ALTs) and attention-deficit/hyperactivity disorder (ADHD)-like traits (ADHLTs), may increase vulnerability to psychological distress. However, little is known about how these traits relate to burnout, depression and anxiety, and empathy among junior residents.

**Methods:**

In this cross-sectional study, 148 junior residents from two teaching hospitals in Japan completed validated measures of ALTs (21-item Japanese version of the Autism-Spectrum Quotient), ADHLTs (Adult ADHD Self-Report Scale Screener), burnout (Maslach Burnout Inventory), depression and anxiety (Hospital Anxiety and Depression Scale), physician–patient empathy (Jefferson Scale of Physician Empathy), and psychological flexibility and inflexibility processes (Valuing Questionnaire, Cognitive Fusion Questionnaire-7, and Work-related Acceptance and Action Questionnaire). Associations were examined using multivariable logistic and linear regression analyses. Exploratory statistical mediation analyses using structural equation modeling examined indirect associations through psychological flexibility and inflexibility processes.

**Results:**

The prevalence of ALTs and ADHLTs was 23.6% for each trait. ALTs were associated with lower personal accomplishment, a burnout dimension; higher depression and anxiety; and lower physician–patient empathy. ADHLTs were associated with greater emotional exhaustion, another burnout dimension. In exploratory statistical mediation analyses, progress toward values, a core process of psychological flexibility, showed a significant indirect association between ALTs and personal accomplishment, and the direct association was attenuated and no longer statistically significant after including the process variables. Significant indirect associations through progress toward values were also observed for the associations of ALT with depression and anxiety and empathy. Cognitive fusion, a core process of psychological inflexibility, showed a significant indirect association between ADHLTs and emotional exhaustion. Overall, neurodevelopmental traits were associated with distinct patterns of psychological functioning, suggesting variability in both vulnerability and adaptive processes.

**Discussion:**

Neurodevelopmental traits such as ALTs and ADHLTs were significantly associated with burnout dimensions, depression and anxiety, and physician–patient empathy among junior residents. Psychological flexibility and inflexibility processes, particularly progress toward values and cognitive fusion, may be relevant to these associations. Process-based support strategies may warrant further investigation for residents with elevated neurodevelopmental traits.

**Clinical trial registration:**

https://center6.umin.ac.jp/cgi-open-bin/ctr_e/ctr_view.cgi?recptno=R00005, identifier UMIN000046897.

## 1 Introduction

Burnout among physicians is a well-recognized problem, with prevalence rates estimated at approximately 40% (1), and similar findings have been reported in Japan (2). Prevalence rates among medical students and junior residents are also high and considerably exceed those observed in age-matched samples from the general population (3, 4). A previous study focused on medical students in clinical clerkships also reported comparable burnout rates (5). Burnout has been linked to mental health problems, including depression and substance use disorders, and it negatively affects both patient care and healthcare systems (6–9). Among medical trainees, burnout also has physical and cognitive consequences, including sleep disturbances, psychosomatic symptoms, and difficulties with memory and concentration, which may further increase emotional burden (10, 11). These consequences are clinically important because physician burnout has been linked to a higher likelihood of medical errors and reduced quality of care.

Although the lifetime prevalence of depression among physicians is estimated at 25%–30%, comparable to that in other occupational groups (12, 13), the standardized mortality ratio for suicide among physicians is higher, at 1.44 (14). Female physicians, in particular, are at increased risk of both burnout and suicide (9, 15, 16). Among psychiatric disorders other than depression, generalized anxiety disorder affects approximately 24% of physicians, while post-traumatic stress disorder occurs in an estimated 4%–16% (17, 18).

Autism is a neurodevelopmental disorder characterized by difficulties in reciprocal social interaction, social communication, and repetitive patterns of behavior. It is associated with atypical empathy processes and difficulties in understanding and predicting others’ intentions, actions, and emotions (19). Autism is increasingly conceptualized as existing along a continuum from neurotypical development to classical autism. From this dimensional perspective, subthreshold autistic traits, termed autistic-like traits (ALTs), have been identified not only in relatives of individuals with autism but also in the general population (20). Meanwhile, attention-deficit/hyperactivity disorder (ADHD) is a neurodevelopmental disorder defined by persistent symptoms of inattention and/or hyperactivity-impulsivity that impair functioning across two or more settings. ADHD-like traits (ADHLTs) are likewise considered to exist along a continuum that includes neurotypical individuals. Evidence suggests that ADHLTs are associated with internalizing symptoms, including anxiety and depression, particularly in the presence of ALTs (21–23). This suggests that ADHLTs may contribute to psychological vulnerability in ways that partly overlap with, but are not identical to, those related to ALTs. Although some individuals with subthreshold neurodevelopmental traits demonstrate strong academic or professional functioning (24), favorable outcomes cannot be assumed (21). A study focused on pharmacists in Japan reported ALT and ADHLT prevalence rates of 5.5% and 11.6%, respectively, and found that both traits were significantly associated with psychological distress and burnout (25). A previous study among medical students during clinical clerkships (2019–2020) found elevated ALT and ADHLT rates of 27.8% and 20.5%, respectively, and reported significant correlations of these traits with depression, anxiety, and lower physician–patient empathy (26). However, little is known about how these traits are associated with multidimensional patterns of psychological functioning, including burnout, depression and anxiety, and empathy, among physicians in early clinical training. Understanding these patterns may help clarify psychological processes associated with distress and functioning in early-career physicians while acknowledging heterogeneity in both vulnerability and adaptive functioning across individuals.

Psychological flexibility is the ability to remain engaged with the present moment and to act in accordance with personally valued goals even in the presence of distressing internal experiences, such as negative thoughts or emotions (27). Core processes include acceptance, cognitive defusion, and progress toward values, all of which have been associated with reduced distress and improved well-being among healthcare workers (28–33). These processes also form the conceptual foundation of acceptance and commitment therapy (ACT).

Although ACT was not examined directly in this study, its theoretical framework highlights the potential importance of psychological flexibility in understanding how neurodevelopmental traits may be related to burnout, depression and anxiety, and empathy. Therefore, this study aimed to:

1. assess the prevalence of ALTs and ADHLTs, burnout, and depression and anxiety among junior residents in Japan;
2. examine whether these traits are associated with burnout and depression and anxiety;
3. explore whether neurodevelopmental traits are associated with empathy toward patients; and
4. explore indirect associations through psychological flexibility and inflexibility processes.

## 2 Methods

### 2.1 Study design and setting

This cross-sectional observational study was conducted at two institutions in Japan: Nagoya City University Hospital, an urban university-affiliated hospital, and Gifu Hospital, a medium-sized psychiatric specialty hospital. Participants were consecutively recruited among junior residents undergoing clinical psychiatry training at these institutions.

### 2.2 Participant recruitment

In Japan, medical graduates must complete a 2-year postgraduate residency program, beginning in April, to practice medicine. This foundational clinical training includes mandatory rotations of at least 24 weeks in internal medicine and 12 weeks in emergency medicine, as well as additional rotations in community medicine, surgery, pediatrics, obstetrics and gynecology, and psychiatry, each with a minimum duration of 4 weeks.

Between April 2022 and March 2025, 153 junior residents who completed psychiatry rotations at Nagoya City University Hospital were invited to participate in an online survey administered through Google Forms. To reach the target sample size, an additional 23 residents who completed psychiatry training at Gifu Hospital between April 2024 and March 2025 were also invited. No prior information was available regarding participants’ neurodevelopmental or mental health diagnoses, including autism, ADHD, anxiety disorders, or depression. No explicit exclusion criteria based on psychiatric or neurodevelopmental diagnoses, current mental health treatment, or pharmacological treatment were applied. This decision was made because the study aimed to examine neurodevelopmental traits and psychological functioning in a consecutive sample of junior residents in real-world postgraduate training settings. However, information on current psychiatric diagnoses or ongoing treatment was not collected. The study followed the STrengthening the Reporting of OBservational studies in Epidemiology (STROBE) guidelines. Ethical approval was obtained from the Institutional Review Board of the Graduate School of Medical Sciences, Nagoya City University. Electronic informed consent was obtained from all participants before their engagement.

### 2.3 Measures

#### 2.3.1 Demographic and occupational information

Demographic and occupational variables included gender, age, marital status, parental status, cohabitation status, part-time employment, and alcohol use (defined as drinking alcohol 3 or more times per week).

#### 2.3.2 Explanatory variables

ALTs were assessed using the 21-item Japanese version of the Autism-Spectrum Quotient (AQ-J-21). The original Autism-Spectrum Quotient (AQ) is a widely used self-report instrument that measures autistic traits in adults with average or above-average intelligence (34). The AQ-J-21, a shortened version of the 50-item Japanese AQ, has shown superior sensitivity and specificity compared with the full version (35). A cut-off score of ≥12, which distinguishes individuals with autism spectrum disorder from community controls, was used to classify participants as having ALTs. Internal consistency of the AQ-J-21 in this study was acceptable (Cronbach’s α = 0.72).

ADHLTs were measured using the Japanese version of the Adult ADHD Self-Report Scale (ASRS) Screener, developed by Kessler et al. and validated in Japanese epidemiological studies (36, 37). The ASRS consists of 2 parts based on criteria of the Diagnostic and Statistical Manual of Mental Disorders, Fourth Edition (DSM-IV): Part A (6 items), known as the ASRS Screener, and Part B (12 items), which includes additional ADHD symptom items. In this study, participants scoring ≥4 on Part A were classified as having ADHLTs. Internal consistency for the ASRS Screener was good (Cronbach’s α = 0.74).

#### 2.3.3 Outcome variables

Burnout was assessed using the Maslach Burnout Inventory (MBI), a 22-item self-report scale widely regarded as the gold standard for measuring occupational burnout (38). Items are rated on a 7-point Likert scale ranging from 0 (never) to 6 (every day). The MBI includes 3 subscales: Emotional Exhaustion (MBI-EE), Depersonalization (MBI-DP), and Personal Accomplishment (MBI-PA), and separate scores were calculated for each. High MBI-EE scores indicate difficulty coping with emotional demands at work. High MBI-DP scores reflect negative or detached attitudes toward patients, whereas low MBI-PA scores indicate diminished feelings of effectiveness and fulfillment in patient care. The MBI has been widely used in physician research (39–41), and the Japanese version has demonstrated good reliability and validity (42). Burnout criteria were defined according to cut-offs used in large-scale US studies: MBI-EE ≥27 for high emotional exhaustion, MBI-DP ≥10 for high depersonalization, and MBI-PA ≤33 for low personal accomplishment. Overall burnout was defined as meeting the threshold for MBI-EE ≥27 and/or MBI-DP ≥10 (3, 40, 43). In the present study, internal consistency was high for all 3 MBI subscales (MBI-EE α = 0.87, MBI-DP α = 0.71, and MBI-PA α = 0.90).

Depression and anxiety symptoms were assessed using the Hospital Anxiety and Depression Scale (HADS), a 14-item screening tool developed for use in clinical populations (44). The HADS has been widely used in studies involving general student populations and medical students (45–47). It contains 2 subscales, anxiety and depression, each with 7 items rated on a 4-point Likert scale. Higher scores indicate greater symptom severity. Validation studies of the Japanese version have confirmed its psychometric properties. Participants with HADS total scores of ≥20 were classified as having clinically significant depressive and anxiety symptoms, based on established diagnostic thresholds in a validation study of the Japanese version of HADS (48). This total-score threshold was used because the present study aimed to screen for overall psychological distress characterized by depressive and anxiety symptoms, rather than to establish separate clinical diagnoses of anxiety or depressive disorders. Internal consistency was acceptable (Cronbach’s α = 0.82).

Physician–patient empathy was assessed using the Jefferson Scale of Physician Empathy (JSPE), a 20-item self-report instrument with 3 components: perspective taking, compassionate care, and standing in the patient’s shoes. The reliability and validity of both the original and Japanese versions have been confirmed (49, 50). In this study, internal consistency for the JSPE was high (Cronbach’s α = 0.86).

#### 2.3.4 Process variables

Psychological flexibility was measured using 3 instruments: the Valuing Questionnaire (VQ), the Cognitive Fusion Questionnaire-7 (CFQ-7), and the Work-related Acceptance and Action Questionnaire (WAAQ). The VQ is a 10-item scale assessing values-based action and obstruction and includes 2 subscales: progress toward a valued life (VQ-P) and obstruction due to experiential avoidance (VQ-O). Each item is rated on a 7-point Likert scale. Higher VQ-P scores indicate greater engagement in valued living, whereas higher VQ-O scores indicate greater interference with valued living due to avoidance of unpleasant experiences (51). The CFQ-7 is a 7-item instrument that measures cognitive fusion, or the extent to which individuals are entangled with their thoughts (52). Higher scores indicate greater cognitive fusion. The WAAQ is a 7-item scale that measures psychological flexibility specifically in the workplace (53). Higher scores indicate greater flexibility in pursuing valued goals despite internal discomfort. All 3 measures have validated Japanese versions (54–56). The VQ was selected to assess value-consistent behavior and experiential avoidance, whereas the CFQ-7 was included to assess cognitive fusion, a process theoretically relevant to distress and burnout. The WAAQ was chosen to capture experiential avoidance in the context of occupational burnout. In this study, internal consistency was acceptable for all scales except the VQ-O subscale (CFQ-7 α = 0.93, VQ-P α = 0.87, VQ-O α = 0.69, and WAAQ α = 0.87).

### 2.4 Data collection

Data were collected anonymously through an online survey using Google Forms. Surveys were administered during break periods in psychiatry rotations. The survey introduction explained the study purpose, procedures, risks, and benefits and emphasized voluntary participation. Participants provided electronic informed consent before proceeding. No personally identifiable information was collected.

### 2.5 Sample size

The required sample size was determined *a priori*. Following Peduzzi et al., at least 10 outcome events per predictor variable were required for logistic regression analyses (57). Four predictor variables were included: age, gender, ALTs, and ADHLTs. Assuming a prevalence of 25%–30% for severe burnout or clinically significant depressive and anxiety symptoms, approximately 40 cases with the outcome were needed. Thus, a total sample size of 130–160 was considered sufficient.

### 2.6 Statistical analysis

The distributions of continuous variables were examined using visual inspection and the Shapiro–Wilk test. Because several variables showed departures from normality, continuous variables were summarized using medians and interquartile ranges (IQRs), and categorical variables were summarized as numbers and percentages. Bivariate associations between explanatory, outcome, and process variables were examined using Spearman’s rank correlation coefficients. To assess the independent associations between explanatory and outcome variables, multivariable models were constructed while adjusting for age and sex as predefined covariates. Age and sex were selected *a priori* as potential confounders based on prior literature and conceptual considerations regarding confounding control (58). For binary outcomes, multivariable logistic regression analyses were conducted. Further, we tested whether ASRS Screener scores moderated the associations between AQ-J-21 scores and the outcomes. Mean-centered AQ-J-21 and ASRS Screener scores, along with their interaction term (AQ-J-21 × ASRS Screener), were entered into logistic regression models for each binary outcome: high emotional exhaustion, high depersonalization, low personal accomplishment, overall burnout, and high depressive and anxiety symptoms, adjusting for age and sex. Multicollinearity was assessed using variance inflation factors (VIFs), and model fit was evaluated using Nagelkerke R². For the continuous outcome of empathy, measured by the JSPE, multiple linear regression was performed using the same interaction term and covariates. Model fit was evaluated using multiple R². When the interaction term was statistically significant, simple slopes analyses were conducted to estimate the effect of AQ-J-21 at ±1 SD of the ASRS Screener. Results were reported as odds ratios (ORs), 95% confidence intervals (CIs), and p-values for binary outcomes, and as unstandardized coefficients for the continuous outcome.

Sensitivity analyses were also conducted by dichotomizing AQ-J-21 and ASRS Screener scores using established cut-off values to assess the robustness of the findings. Because the number of participants meeting the cut-off for high emotional exhaustion was small and the prevalence of low personal accomplishment was high, additional sensitivity analyses were conducted using continuous MBI-EE, MBI-DP, and MBI-PA scores as outcomes. Additionally, HADS-Total, HADS-Anxiety, and HADS-Depression scores were examined as continuous outcomes. These sensitivity models included mean-centered AQ-J-21 scores, mean-centered ASRS Screener scores, and their interaction term; age; and sex as predictors. Residual diagnostics were inspected, and heteroscedasticity-consistent HC3 robust standard errors were additionally calculated.

Exploratory statistical mediation analyses were conducted using observed-variable path models in structural equation modeling (SEM) with Mplus version 8.8 (Muthén & Muthén) to examine indirect associations through psychological flexibility and inflexibility processes. Age and sex were included as covariates by regressing both the process variables and outcome variables on these covariates. For each model, total, direct, total indirect, and specific indirect effects were estimated within the same model. Both unstandardized coefficients and fully standardized coefficients were examined; the latter correspond to STDYX estimates in Mplus, which standardize both predictor and outcome variables. Process variables were selected based on their theoretical relevance to psychological flexibility and inflexibility processes, prior literature linking these processes to burnout and psychological distress, their internal consistency, and their associations with the outcome variables. Therefore, CFQ-7, VQ-P, and WAAQ were examined as process variables. VQ-O was not included in the exploratory statistical mediation models because of its high correlation with WAAQ and its relatively low internal consistency, to reduce redundancy and avoid overparameterization. Residual covariances among process variables were allowed when multiple process variables were included. Because the observed-variable path models were saturated or just-identified with zero degrees of freedom, conventional global model fit indices were reported descriptively but were not used to evaluate model adequacy. The magnitude of indirect effects was estimated using bias-corrected bootstrapping with 1, 000 resamples to calculate 95% CIs (59). Indirect effects were considered statistically significant when the 95% CI did not include zero. Given the cross-sectional design, these analyses were not intended to establish temporal or causal mediation.

Statistical analyses other than SEM were conducted using EZR, a graphical interface for R version 3.6.1. A 2-tailed p-value of <0.05 was considered statistically significant. Given the exploratory nature of the study and the multiple outcomes and models examined, no formal correction for multiple comparisons was applied. Therefore, p-values, particularly those close to the significance threshold, were interpreted cautiously.

## 3 Results

### 3.1 Sociodemographic characteristics

Of the 176 junior residents invited to participate, 148 (84.1%) completed the survey (**Table 1**). Of them, 78 (52.7%) were male and 70 (47.3%) were female. The median age was 27.0 years (IQR, 26.0-27.3). The prevalence of ALTs and ADHLTs was 23.6% (n = 35, 95% CI, 17.1-31.3) for each trait. Regarding burnout dimensions, the prevalence of high emotional exhaustion (MBI-EE) was 6.8% (n = 10, 95% CI, 3.3-12.1), high depersonalization (MBI-DP) was 41.9% (n = 62, 95% CI, 33.8-50.3), and low personal accomplishment (MBI-PA) was 81.1% (n = 120, 95% CI, 73.8-87.0). Overall burnout was observed in 44.6% of the sample (n = 66, 95% CI, 36.4-53.0), whereas clinically significant depressive and anxiety symptoms were observed in 18.2% (n = 27, 95% CI, 12.4-25.4).

**Table 1:** Demographic characteristics and descriptive data.

	Total number (n=148)
Variable	Number (%)
Sex (male)	78 (52.7)
Sex (female)	70 (47.3)
ALTs ^a^	35 (23.6)
ADHLTs ^b^	35 (23.6)
High emotional exhaustion^*^	10 (6.8)
High depersonalization^*^	62 (41.9)
Low personal accomplishment^*^	120 (81.1)
Overall burnout^*^	66 (44.6)
High depression/anxiety^**^	27 (18.2)
	Median [IQR^***^]
Age	27.0 [26.0–27.3]
AQ-J-21 ^c^	6.0 [3.0–9.0]
ASRS Screener ^d^	2.0 [1.0–4.0]
MBI-EE ^e^	17.0 [11.0–22.0]
MBI-DP ^f^	9.0 [5.0–13.0]
MBI-PA ^g^	31.0 [27.0–35.0]
HADS-Total ^h^	13.0 [8.0–18.0]
JSPE ^i^	107.0 [101.0–114.0]
CFQ-7 ^j^	20.0 [15.0–26.0]
VQ-P ^k^	19.0 [15.0–22.0]
VQ-O ^l^	13.0 [10.0–16.0]
WAAQ ^m^	34.0 [29.0–39.0]

^*^ MBI-EE score of ≥27 was defined as high emotional exhaustion, MBI-DP score of ≥10 as high depersonalization, MBI-PA score of ≤33 as a low degree of personal accomplishment, and MBI-EE score of ≥27 and/or MBI-DP score of ≥10 as overall burnout.

^**^ A HADS total score of ≥20 was classified as clinically significant depressive and anxiety symptoms.

^***^ IQR, interquartile range.

^a^ ALTs, autistic-like traits; ^b^ADHLTs, attention-deficit/hyperactivity disorder-like traits; ^c^AQ-J-21, 21-item Japanese version of the Autism-Spectrum Quotient; ^d^ASRS Screener, Adult ADHD Self-Report Scale Screener; ^e^MBI-EE, Maslach Burnout Inventory, emotional exhaustion; ^f^MBI-DP: Maslach Burnout Inventory, depersonalization; ^g^MBI-PA, Maslach Burnout Inventory, personal accomplishment; ^h^HADS-Total, Hospital Anxiety and Depression Scale, total score; ^i^JSPE, Jefferson Scale of Physician Empathy; ^j^CFQ-7, Cognitive Fusion Questionnaire-7; ^k^VQ-P, Valuing Questionnaire, progress; ^l^VQ-O, Valuing Questionnaire, obstruction; ^m^WAAQ, Work-related Acceptance and Action Questionnaire.

### 3.2 Correlation analysis

Bivariate correlations showed that ALTs (AQ-J-21) were positively associated with ADHLTs (ASRS Screener), depressive and anxiety symptoms (HADS total), and cognitive fusion (CFQ-7), and negatively associated with personal accomplishment (MBI-PA), physician–patient empathy (JSPE), values progress (VQ-P), and work-related psychological flexibility (WAAQ). ASRS Screener scores were positively associated with emotional exhaustion (MBI-EE), depressive and anxiety symptoms, cognitive fusion (CFQ-7), and values obstruction (VQ-O), and negatively associated with values progress (VQ-P). Empathy (JSPE) was positively associated with personal accomplishment and values progress, whereas depressive and anxiety symptoms were negatively associated with empathy, work-related psychological flexibility, and values progress. Complete correlation coefficients are presented in **Table 2**.

**Table 2:** Results of correlation analysis.

	1	2	3	4	5	6	7	8	9	10	11
1. AQ-J-21 a	1.00										
2. ASRS Screener b	0.20 ^*^	1.00									
3. MBI-EE c	0.12	0.24 ^**^	1.00								
4. MBI-DP d	0.08	0.02	-0.04	1.00							
5. MBI-PA e	-0.23 ^**^	0.04	0.24 ^**^	-0.08	1.00						
6. HADS-Total f	0.38 ^**^	0.27 ^**^	0.23 ^**^	0.08	-0.17 ^*^	1.00					
7. JSPE g	-0.33 ^**^	-0.06	-0.04	-0.10	0.38 ^**^	-0.46 ^**^	1.00				
8. CFQ-7 h	0.22 ^**^	0.28 ^**^	0.31 ^**^	0.18 ^*^	0.02	0.56 ^**^	-0.13	1.00			
9. VQ-P ^i^	-0.33 ^**^	-0.17 ^*^	-0.12	0.09	0.42 ^**^	-0.51 ^**^	0.38 ^**^	-0.21 ^**^	1.00		
10. VQ-O j	0.09	0.32 ^**^	0.23 ^**^	0.11	0.06	0.36 ^**^	-0.10	0.59 ^**^	-0.05	1.00	
11. WAAQ k	-0.18 ^*^	-0.12	-0.11	0.14	0.17 ^*^	-0.38 ^**^	0.08	-0.21 ^*^	0.30 ^**^	-0.10	1.00

^*^ *p* < 0.05.

^**^ *p* < 0.01.

^a^ AQ-J-21, 21-item Japanese version of the Autism-Spectrum Quotient; ^b^ASRS Screener, Adult ADHD Self-Report Scale Screener; ^c^MBI-EE, Maslach Burnout Inventory, emotional exhaustion; ^d^MBI-DP, Maslach Burnout Inventory, depersonalization; ^e^MBI-PA, Maslach Burnout Inventory, personal accomplishment; ^f^HADS-Total, Hospital Anxiety and Depression Scale, total score; ^g^JSPE, Jefferson Scale of Physician Empathy; ^h^CFQ-7, Cognitive Fusion Questionnaire-7; ^i^VQ-P, Valuing Questionnaire, progress; ^j^VQ-O, Valuing Questionnaire, obstruction; ^k^WAAQ, Work-related Acceptance and Action Questionnaire.

### 3.3 Logistic regression analysis

In multivariable logistic regression analyses adjusted for age and sex, higher AQ-J-21 scores were significantly associated with low personal accomplishment and clinically significant depressive and anxiety symptoms. Higher ASRS Screener scores were significantly associated with high emotional exhaustion. Depersonalization was not associated with either trait but was significantly associated with younger age (**Table 3A**).

**Table 3A:** Binary logistic regression with burnout and depression/anxiety as outcome variables.

		95% CI^*^		
Variables	OR	Lower	Upper	*p*-value
High emotional exhaustion				
Age	1.19	0.975	1.45	0.087
Sex (male)	0.527	0.127	2.19	0.379
AQ-J-21	1.04	0.866	1.26	0.649
**ASRS Screener**	**1.79**	**1.1**	**2.91**	**0.019**
High depersonalization				
**Age**	**0.837**	**0.705**	**0.993**	**0.041**
Sex (male)	1.11	0.56	2.2	0.764
AQ-J-21	1.01	0.919	1.12	0.783
ASRS Screener	0.959	0.764	1.2	0.715
Low personal accomplishment				
Age	1.07	0.883	1.29	0.501
Sex (male)	0.74	0.309	1.77	0.499
**AQ-J-21**	**1.21**	**1.05**	**1.39**	**0.009**
ASRS Screener	1.02	0.764	1.37	0.886
Overall burnout				
Age	0.911	0.788	1.05	0.204
Sex (male)	0.911	0.466	1.78	0.786
AQ-J-21	1.03	0.939	1.14	0.493
ASRS Screener	0.986	0.79	1.23	0.904
High depression/anxiety				
Age	1.02	0.852	1.21	0.868
Sex (male)	1.07	0.441	2.61	0.875
**AQ-J-21**	**1.15**	**1.01**	**1.3**	**0.030**
ASRS Screener	1.19	0.894	1.59	0.232

^*^ CI: confidence interval.

^a^ AQ-J-21, 21-item Japanese version of the Autism-Spectrum Quotient; ^b^ASRS Screener, Adult ADHD Self-Report Scale Screener.

For values in bold, statistical significance set at *p*  < 0.05.

In models including the AQ-J-21 × ASRS Screener interaction term, the ASRS Screener score was independently associated with high emotional exhaustion (OR = 1.72, 95% CI, 1.03-2.86, p = 0.037), whereas the interaction term was not significant (p = 0.166; Nagelkerke R² = 0.195). Because the number of participants with high emotional exhaustion was small and the prevalence of low personal accomplishment was high, we conducted sensitivity analyses using continuous MBI subscale scores as outcomes. ASRS Screener scores were positively associated with MBI-EE scores (B = 1.40, 95% CI, 0.41 to 2.38, p = 0.006), whereas AQ-J-21 scores and the AQ-J-21 × ASRS Screener interaction were not significant. AQ-J-21 scores were negatively associated with MBI-PA scores (B = -0.90, 95% CI, -1.42 to -0.37, p = 0.001), whereas ASRS Screener scores were not significant. The AQ-J-21 × ASRS Screener interaction for MBI-PA did not reach statistical significance (B = 0.33, 95% CI, -0.005 to 0.67, p = 0.053). No significant associations with MBI-DP scores were observed for AQ-J-21, ASRS Screener, or their interaction term. The interaction terms were not statistically significant in any of the continuous MBI subscale models. VIF values indicated no problematic multicollinearity, and HC3 robust standard errors yielded the same overall conclusion (**Supplementary Table 6**). For high depersonalization, younger age was associated with higher odds (OR = 0.84, p = 0.050), while the AQ-J-21, ASRS Screener, and their interaction were not significant (Nagelkerke R² = 0.055). For low personal accomplishment, the AQ-J-21 had a positive association (OR = 1.27, p = 0.003), and the AQ-J-21 × ASRS Screener interaction was significant (OR = 0.88, p = 0.0048; Nagelkerke R² = 0.172), suggesting that higher ASRS Screener scores attenuated the association between AQ-J-21 and low personal accomplishment. For clinically significant depressive and anxiety symptoms, the AQ-J-21 had a positive association (OR = 1.22, p = 0.0068), and the interaction with the ASRS Screener was significant (OR = 0.91, p = 0.045; Nagelkerke R² = 0.133), again suggesting attenuation of the AQ-J-21-outcome association at higher ASRS Screener levels. No significant effects were observed for overall burnout (Nagelkerke R² = 0.023). VIF values (1.0-1.8) indicated no problematic multicollinearity (**Table 3B**).

**Table 3B:** Binary logistic regression with burnout and depression/anxiety as outcome variables, with the AQ-J-21 × ASRS screener interaction.

		95% CI^*^		
Variables	OR	Lower	Upper	*p*-value
High emotional exhaustion				
Age	1.15	0.92	1.42	0.212
Sex (male)	0.55	0.12	2.39	0.423
AQ-J-21_c	0.95	0.75	1.20	0.645
**ASRS Screener_c**	**1.72**	**1.03**	**2.86**	**0.037**
AQ-J-21_c × ASRS_c	1.09	0.96	1.24	0.166
High depersonalization				
**Age**	**0.84**	**0.71**	**1.00**	**0.050**
Sex (male)	1.07	0.54	2.14	0.840
AQ-J-21_c	1.02	0.92	1.13	0.699
ASRS Screener_c	0.96	0.77	1.21	0.748
AQ-J-21_c × ASRS_c	0.97	0.91	1.04	0.412
Low personal accomplishment				
Age	1.11	0.91	1.36	0.303
Sex (male)	0.65	0.26	1.63	0.359
**AQ-J-21_c**	**1.27**	**1.08**	**1.49**	**0.003**
ASRS Screener_c	0.93	0.68	1.27	0.654
**AQ-J-21_c × ASRS_c**	**0.88**	**0.80**	**0.96**	**0.005**
Overall burnout				
Age	0.91	0.78	1.05	0.191
Sex (male)	0.93	0.47	1.82	0.821
AQ-J-21_c	1.03	0.93	1.14	0.545
ASRS Screener_c	0.98	0.79	1.23	0.875
AQ-J-21_c × ASRS_c	1.01	0.95	1.08	0.662
High depression/anxiety				
Age	1.05	0.88	1.25	0.591
Sex (male)	0.96	0.39	2.36	0.923
**AQ-J-21_c**	**1.22**	**1.06**	**1.41**	**0.007**
ASRS Screener_c	1.35	0.98	1.86	0.067
**AQ-J-21_c × ASRS_c**	**0.91**	**0.83**	**1.00**	**0.045**

^*^ CI, confidence interval.

^a^ AQ-J-21, 21-item Japanese version of the Autism-Spectrum Quotient; ^b^ASRS Screener, Adult ADHD Self-Report Scale Screener. Variables with the suffix “_c” indicate mean-centered values. Interaction terms (e.g., AQ-J-21_c × ASRS Screener_c) were computed from the centered variables.

For values in bold, statistical significance set at *p*  <0.05.

In sensitivity analyses using continuous HADS-Total, HADS-Anxiety, and HADS-Depression scores as outcomes, AQ-J-21 scores were consistently positively associated with all three outcomes. ASRS Screener scores were also positively associated with HADS-Total and HADS-Depression scores and showed a borderline association with HADS-Anxiety when HC3 robust standard errors were applied. The interaction terms were not statistically significant in any of these models (**Supplementary Table 7**).

### 3.4 Multiple linear regression analysis

In multiple linear regression analysis adjusted for age and sex, higher AQ-J-21 scores were significantly associated with lower physician–patient empathy as measured by JSPE scores (**Table 4A**). The interaction between the AQ-J-21 and ASRS Screener on JSPE scores was significant (B = 0.514, p = 0.020; **Table 4B**). The model accounted for approximately 15% of the variance in JSPE scores (multiple R² = 0.151; adjusted R² = 0.121). VIF values (1.0-1.8) indicated no problematic multicollinearity.

**Table 4A:** Linear multiple regression with physician–patient empathy as the outcome variable.

		95% CI^*^				
Variables	B	Lower	Upper	t	*p*-value	Multiple R^2^
JSPE ^a^						
Constant	106.400	80.604	132.196	8.153	0.000	
Age	0.245	-0.677	1.167	0.525	0.601	
Sex (male)	2.704	-1.947	7.354	1.149	0.252	
**AQ-J-21 ^b^**	**-1.428**	**-2.096**	**-0.759**	**-4.223**	**0.000**	
ASRS Screener ^c^	-0.052	-1.595	1.492	-0.066	0.947	0.118

^*^ CI, confidence interval.

^a^ JSPE, Jefferson Scale of Physician Empathy; ^b^AQ-J-21, 21-item Japanese version of the Autism-Spectrum Quotient; ^c^ASRS Screener, Adult ADHD Self-Report Scale Screener.

For values in bold, statistical significance set at *p*  < 0.05.

**Table 4B:** Linear multiple regression with physician–patient empathy as the outcome variable, including the AQ-J-21 × ASRS screener interaction.

		95% CI^*^				
Variables	B	Lower	Upper	t	*p*-value	Multiple R^2^
JSPE ^a^								
Constant	96.317	71.470	121.164	7.657	0.000	
Age	0.121	-0.786	1.027	0.261	0.795	
Sex (male)	3.260	-1.304	7.823	1.400	0.164	
**AQ-J-21_c ^b^**	**-1.564**	**-2.226**	**-0.902**	**-4.629**	**0.000**	
ASRS-S_c ^c^	-0.186	-1.697	1.325	-0.241	0.810	
**AQ-J-21_c × ASRS_c**	**0.514**	**0.086**	**0.942**	**2.353**	**0.020**	**0.151**

^*^ CI, confidence interval.

^a^ JSPE, Jefferson Scale of Physician Empathy; ^b^AQ-J-21, 21-item Japanese version of the Autism-Spectrum Quotient; ^c^ASRS Screener, Adult ADHD Self-Report Scale Screener. Variables with the suffix “_c” indicate mean-centered values. Interaction terms (e.g., AQ-J-21_c × ASRS Screener_c) were computed from the centered variables.

For values in bold, statistical significance set at *p*  < 0.05.

### 3.5 Interaction probing (simple slopes)

Simple slopes of the AQ-J-21 at -1 SD, the mean, and +1 SD of the ASRS Screener are presented in **Supplementary Table 1**–**3**. For low personal accomplishment, high depressive and anxiety symptoms, and JSPE scores, the slopes were significant at lower and mean levels of the ASRS Screener, consistent with the significant AQ-J-21 × ASRS interaction observed in the main models.

### 3.6 Interaction probing (sensitivity analyses)

In the 4-group sensitivity analysis (**Supplementary Table 4**), all 3 higher-trait groups had significantly greater odds of high depressive and anxiety symptoms than the low ALT/low ADHLT group (adjusted ORs, 4.85-6.88; p = 0.028-0.002). For low personal accomplishment, estimates for the high ALT/low ADHLT and low ALT/high ADHLT groups were above 1.0, but their CIs included 1. Given the relatively small size of the high ALT/high ADHLT subgroup (n = 13), estimates for this group should be interpreted with caution. For empathy (JSPE; **Supplementary Table 5**), both the high ALT/high ADHLT group (B = -8.49, 95% CI, -16.75 to -0.22; p = 0.044) and the high ALT/low ADHLT group (B = -13.36, 95% CI, -20.14 to -6.58; p < 0.001) had significantly lower scores than the low ALT/low ADHLT group, whereas the low ALT/high ADHLT group did not differ significantly from the reference group. This pattern of group differences was consistent with the interaction effect observed in the main regression model.

### 3.7 Exploratory statistical mediation analysis

Exploratory statistical mediation analyses were conducted to examine indirect associations through psychological flexibility and inflexibility processes between neurodevelopmental traits (ALTs or ADHLTs) and psychological outcomes (MBI-EE, MBI-PA, HADS total, and JSPE scores). Four age- and sex-adjusted observed-variable path models were examined. CFQ-7, VQ-P, and WAAQ were included as process variables, whereas VQ-O was not included because of its high correlation with WAAQ and its relatively low internal consistency, as described in the Statistical Analysis section. Detailed model fit indices; standardized path coefficients; and total, direct, and indirect effects are shown in **Supplementary Table 8**.

**Figure 1:**
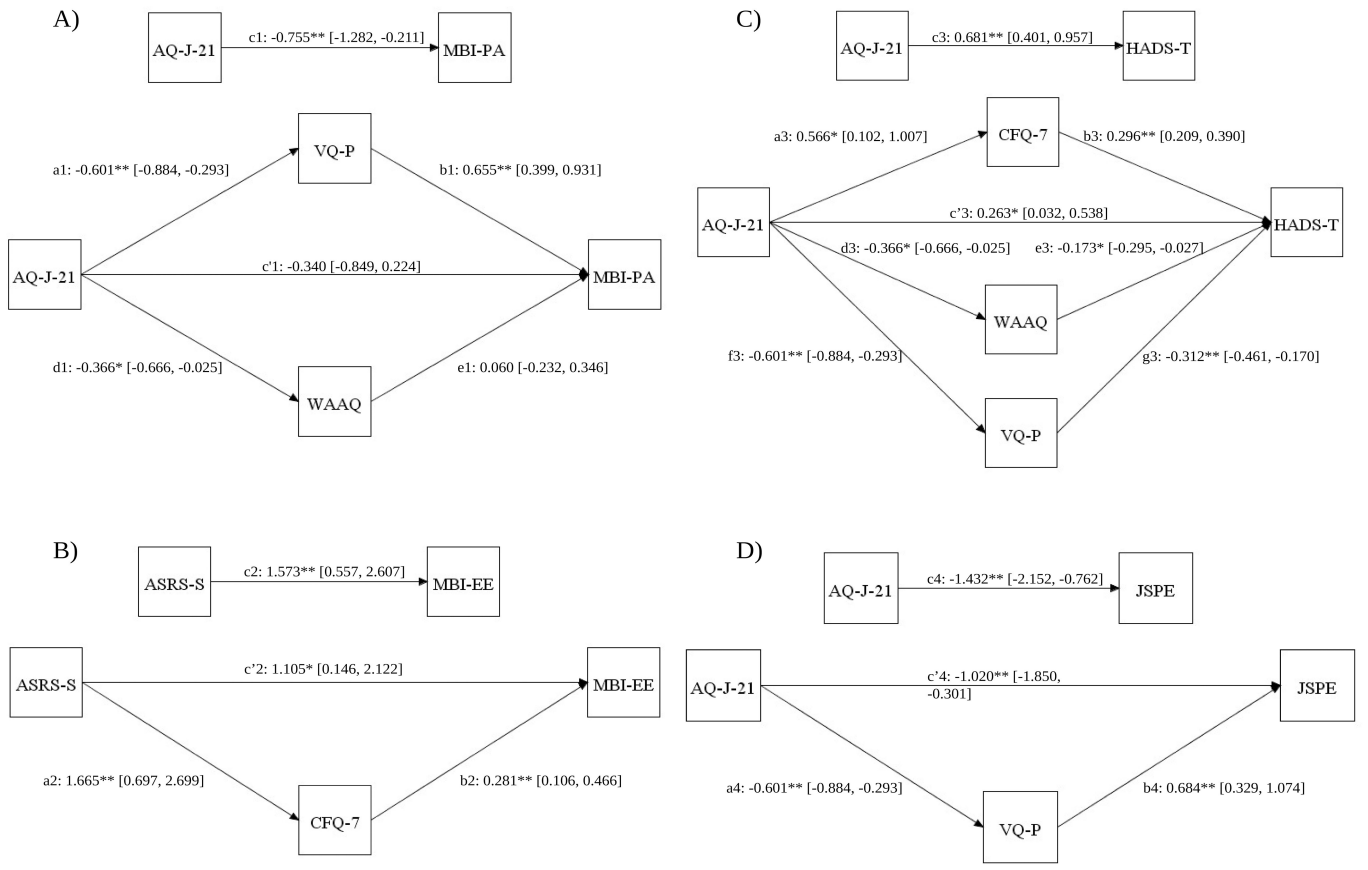
Age- and sex-adjusted exploratory statistical mediation models. Values indicate unstandardized path coefficients, with bias-corrected bootstrap 95% confidence intervals shown in square brackets. All models were adjusted for age and sex by regressing both the process variables and outcome variables on these covariates. Covariate paths are omitted from the figure for visual clarity. Residual covariances among process variables were allowed when multiple process variables were included. Detailed model fit indices; standardized path coefficients; and total, direct, and indirect effects are presented in **Supplementary Table 8**. **(A)** Path c1 represents the total effect of AQ-J-21 on MBI-PA before accounting for any mediators. Path a1 represents the effect of AQ-J-21 on VQ-P. Path b1 represents the effect of VQ-P on MBI-PA in the mediation model. Path d1 represents he effect of AQ-J-21 on WAAQ, and path e1 represents the effect of WAAQ on MBI-PA in the mediation model. The direct effect of AQ-J-21 on MBI-PA after including the mediators is represented by path c′1 and was estimated directly within the SEM model. The specific indirect effects were estimated as the products of the corresponding paths, a1 × b1 and d1 × e1. The model explained 19.4% of the variance in MBI-PA (R² = 0.194). **(B)** Path c2 represents the total effect of the ASRS Screener (ASRS-S) on MBI-EE. Path a2 represents the effect of ASRS-S on CFQ-7. Path b2 represents the effect of CFQ-7 on MBI-EE in the mediation model. The direct effect of ASRS-S on MBI-EE after including CFQ-7 is represented by path c′2 and was estimated directly within the SEM model. The specific indirect effect was estimated as a2 × b2. The model explained 17.0% of the variance in MBI-EE (R² = 0.170). **(C)** Path c3 represents the total effect of AQ-J-21 on HADS-Total (HADS-T). Path a3 represents the effect of AQ-J-21 on CFQ-7, and path b3 represents the effect of CFQ-7 on HADS-T in the mediation model. Path d3 represents the effect of AQ-J-21 on WAAQ, and path e3 represents the effect of WAAQ on HADS-T in the mediation model. Path f3 represents the effect of AQ-J-21 on VQ-P, and path g3 represents the effect of VQ-P on HADS-T in the mediation model. The direct effect of AQ-J-21 on HADS-T after including the mediators is represented by path c′3 and was estimated directly within the SEM model. The specific indirect effects were estimated as a3 × b3, d3 × e3, and f3 × g3. The model explained 52.1% of the variance in HADS-T (R² = 0.521). **(D)** Path c4 represents the total effect of AQ-J-21 on JSPE. Path a4 represents the effect of AQ-J-21 on VQ-P. Path b4 represents the effect of VQ-P on JSPE in the mediation model. The direct effect of AQ-J-21 on JSPE after including VQ-P is represented by path c′4 and was estimated directly within the SEM model. The specific indirect effect was estimated as a4 × b4. The model explained 19.8% of the variance in JSPE (R² = 0.198). Interaction analyses and mediation analyses were conducted separately; therefore, the AQ-J-21 × ASRS Screener interaction models reported in the regression analyses are not shown in this figure. *p <0.05, **p <0.01. AQ-J-21, 21-item Japanese version of the Autism-Spectrum Quotient; ASRS-S, Adult ADHD Self-Report Scale Screener; CFQ-7, Cognitive Fusion Questionnaire-7; HADS-T, Hospital Anxiety and Depression Scale total score; JSPE, Jefferson Scale of Physician Empathy; MBI-EE, Maslach Burnout Inventory, emotional exhaustion subscale; MBI-PA, Maslach Burnout Inventory, personal accomplishment subscale; SEM, structural equation modeling; VQ-P, Valuing Questionnaire, progress subscale; WAAQ, Work-related Acceptance and Action Questionnaire.

­ For MBI-PA scores, AQ-J-21 scores were significantly negatively associated with MBI-PA scores in the model before including process variables (path coefficient c1, p < 0.01). In the exploratory statistical mediation model, a significant indirect association was observed through VQ-P (a1 × b1), as the bootstrap 95% CI did not include zero. After including VQ-P and WAAQ in the model, the direct association between AQ-J-21 scores and MBI-PA scores was attenuated and was no longer statistically significant (c′1, p > 0.05; **Figure 1A**).

­ For MBI-EE scores, ASRS Screener scores were significantly positively associated with MBI-EE scores in the model before including CFQ-7 (c2, p < 0.01). A significant indirect association was observed through CFQ-7 (a2 × b2), as the bootstrap 95% CI did not include zero. The direct association between ASRS Screener scores and MBI-EE scores remained statistically significant after including CFQ-7 (c′2, p < 0.05; **Figure 1B**).

­ For HADS total scores, AQ-J-21 scores were significantly positively associated with HADS total scores in the model before including process variables (c3, p < 0.01). In the exploratory statistical mediation model, significant indirect associations were observed through CFQ-7 (a3 × b3) and VQ-P (f3 × g3), as the bootstrap 95% CIs did not include zero. A smaller indirect association through WAAQ (d3 × e3) was also observed, although this pathway should be interpreted cautiously. The direct association between AQ-J-21 scores and HADS total scores remained statistically significant after including these process variables (c′3, p < 0.05; **Figure 1C**).

­ For JSPE scores, AQ-J-21 scores were significantly negatively associated with JSPE scores in the model before including VQ-P (c4, p < 0.01). A significant indirect association was observed through VQ-P (a4 × b4), as the bootstrap 95% CI did not include zero. The direct association between AQ-J-21 scores and JSPE scores also remained statistically significant after including VQ-P (c′4, p < 0.05; **Figure 1D**).

## 4 Discussion

### 4.1 Main findings

This study examined associations between neurodevelopmental traits and multiple dimensions of psychological functioning among junior residents, including burnout, depressive and anxiety symptoms, and empathy. The prevalence of ALTs and ADHLTs was 23.6% for each trait, similar to previous findings among medical students in clinical clerkships (5, 26). More than 80% of participants reported low personal accomplishment. By contrast, the prevalence of high emotional exhaustion was low, whereas that of depersonalization was relatively high, and that of depressive and anxiety symptoms was below 20%, differing from earlier reports. Neurodevelopmental traits may be linked to these patterns through several possible cognitive and affective processes. Autistic traits are commonly associated with difficulties in social reciprocity, perspective-taking, and emotion recognition, which may increase vulnerability to interpersonal stress in clinical settings. By contrast, ADHD traits are associated with executive functioning difficulties and problems with emotion regulation, which may undermine coping resources and increase distress under demanding conditions. Evidence also suggests that ADHLTs are associated with internalizing symptoms, including anxiety and depression, particularly when co-occurring with ALTs (21–23). These possible processes provide a theoretical basis for the observed patterns of association and suggest that neurodevelopmental traits may relate to different patterns of vulnerability and adaptive functioning rather than show uniform associations across outcomes. This insight underscores the importance of considering combinations of traits when examining physician well-being. Although this study was observational in scope, the use of multivariable models and exploratory statistical mediation analyses may help generate hypotheses about cognitive and affective processes associated with these patterns and highlight heterogeneity in how neurodevelopmental traits relate to both vulnerability and adaptive functioning.

### 4.2 Burnout and psychological distress among junior residents

A possible explanation for the lower prevalence of emotional exhaustion is the 2019 amendment to Japan’s Labor Standards Act, which introduced legal limits on overtime work. These regulations were extended to physicians in 2024 (60). Although reductions in emotional exhaustion and depressive and anxiety symptoms would be encouraging, the high prevalence of depersonalization and low personal accomplishment, resulting in an overall burnout rate exceeding 40%, remains concerning. Similar trends have been reported in the United States, where duty-hour restrictions reduced emotional exhaustion but had mixed effects on patient care and resident well-being (61). In Japan, the super-rotation system introduced in 2005 mandates brief rotations across multiple specialties. This system may limit opportunities for in-depth learning and hands-on clinical experience (62), potentially contributing to low personal accomplishment. Moreover, this study was conducted in the post-acute phase of the COVID-19 pandemic, whereas earlier studies were conducted before or during its acute phase. A recent longitudinal study in Spain found that although emotional exhaustion peaked during the acute phase of COVID-19, depersonalization and reduced personal accomplishment became more prominent during the chronic phase (63).

### 4.3 Differential associations of ALTs and ADHLTs with burnout, depression/anxiety, and empathy

After adjustment for age and gender, ALTs were associated with lower personal accomplishment, higher depressive and anxiety symptoms, and lower physician–patient empathy. By contrast, ADHLTs were associated with higher emotional exhaustion. These findings are broadly consistent with previous research among medical students (5, 26). Although depersonalization contributed to the high overall burnout rate, it was not associated with either neurodevelopmental trait and was significantly associated only with younger age. This outcome may reflect more limited coping experiences and greater vulnerability to emotional distancing among younger residents, as observed in post-pandemic healthcare settings (63). The significant AQ-J-21 × ASRS Screener interactions for low personal accomplishment and high depressive and anxiety symptoms suggest an antagonistic pattern in which elevated ADHLTs attenuated the adverse associations of ALTs with these outcomes. However, given the exploratory nature of the interaction analyses and the number of models examined, these findings should be interpreted cautiously and require replication. One possible interpretation is that attentional or motivational characteristics associated with ADHLTs may partially counterbalance the associations of ALTs with self-evaluation and affective symptoms in some contexts. By contrast, emotional exhaustion appeared to be related primarily to ADHLTs, with no moderation by ALTs. These findings underscore the importance of examining combinations of neurodevelopmental traits rather than single traits in isolation. An alternative explanation may relate to features of the measurement tools. The AQ-J-21 includes both positively and negatively worded items, whereas the ASRS Screener consists exclusively of negatively worded items, such as difficulties in attention, organization, or impulsivity. Individuals who endorse more of these items may show greater self-reflection and awareness of their difficulties. Such self-awareness could facilitate more adaptive coping strategies and thereby attenuate the adverse associations of ALTs with depressive and anxiety symptoms and personal accomplishment. This possibility may also help explain why higher ASRS scores were associated with greater empathy, as the ability to recognize one’s own limitations may promote perspective-taking and sensitivity to others’ experiences.

The findings on empathy may also have practical relevance for postgraduate training. Physician–patient empathy has been linked to patient satisfaction, patient enablement, reduced patient anxiety and distress, and better clinical outcomes (64). Lower self-reported empathy among residents with higher ALTs may therefore indicate a need for supportive learning environments that help residents notice their own and patients’ perspectives, reflect on interpersonal cues, and develop flexible communication strategies without pathologizing neurodevelopmental traits. However, because empathy was assessed through self-reporting and patient-level outcomes were not measured, these clinical implications should be interpreted cautiously.

### 4.4 Process-based findings related to ALTs and ADHLTs

In the exploratory statistical mediation analyses, significant indirect associations through progress toward values were observed between ALTs and multiple outcomes. For the association between ALTs and personal accomplishment, the direct association was attenuated and no longer statistically significant after including the process variables. For depressive and anxiety symptoms and physician–patient empathy, significant indirect associations through VQ-P were also observed, while the direct associations remained statistically significant. This pattern is partly consistent with the pattern observed in our previous study of medical students during clinical clerkships, in which VQ-P showed indirect associations in the relationships of ALTs with MBI-PA and HADS scores (5). These findings suggest that reduced progress toward values may be a particularly relevant process for residents with higher ALTs. Consistent engagement in personally meaningful behavior may help support personal accomplishment, empathy, and psychological well-being, although the cross-sectional design precludes conclusions about temporal or causal pathways.

Exploratory statistical mediation analysis also indicated that different components of psychological flexibility and inflexibility may be relevant to ALTs and ADHLTs in different ways. For ALTs, value-based processes such as progress toward values appeared most salient, consistent with the view that social-cognitive challenges in autism spectrum conditions may increase the importance of meaning-oriented engagement for sustaining well-being. By contrast, for ADHLTs, cognitive fusion showed a significant indirect association with emotional exhaustion, suggesting that this process may be particularly relevant to residents with elevated ADHLTs. This finding is consistent with evidence that difficulties in attentional control and set-shifting among individuals with ADHD traits may increase vulnerability to becoming entangled with distressing thoughts. The findings suggest that these processes do not operate uniformly across neurodevelopmental traits but may instead show trait-specific patterns, with implications for tailored support. Furthermore, indirect associations through cognitive fusion and work-related experiential acceptance were also observed for the association between ALTs and depressive and anxiety symptoms, although the WAAQ pathway was smaller and should be interpreted cautiously. Thus, future interventions may need to examine not only value-based action but also cognitive defusion and experiential acceptance as potentially relevant processes for improving mental health in physicians with high ALTs.

A significant indirect association was also observed between ADHLTs and emotional exhaustion through cognitive fusion, while the direct association remained statistically significant. Previous research among Japanese pharmacists similarly reported associations between ADHLTs and burnout (25). Cognitive fusion may be particularly relevant to emotional exhaustion among residents with high ADHLTs. Recent research involving nurses, physicians, and medical students has increasingly identified cognitive fusion as an important factor associated with emotional exhaustion (5, 63, 65, 66). Therefore, future support strategies enhancing cognitive defusion skills—that is, reducing entanglement with distressing thoughts—may be important for supporting the mental health of residents with elevated ALTs and ADHLTs.

### 4.5 Measurement considerations for work-related acceptance

A smaller indirect association through work-related experiential acceptance, assessed by the WAAQ, was also observed for the association between ALTs and depressive and anxiety symptoms, although this pathway should be interpreted cautiously. Cognitive fusion reflects the entanglement with verbal thoughts, whereas experiential acceptance involves the willingness to experience unpleasant internal events, including thoughts, emotions, and impulses, without avoidance (52, 53). Recognizing and articulating emotional experiences may be more difficult than noticing or analyzing cognitive content, and self-report measures may not fully capture this difficulty. Alternatively, the WAAQ, which measures acceptance specifically within workplace contexts, may not accurately reflect experiential acceptance among physicians, whose occupational environments differ qualitatively from those of general workers. The distinctive emotional and ethical demands placed on physicians may limit the applicability of workplace-specific acceptance measures. Given these possibilities, future research could incorporate implicit assessment tools, such as the implicit relational assessment procedure, rather than relying solely on explicit self-report measures, to more accurately capture acceptance processes among physicians (67–69).

### 4.6 Future directions and implications

The current findings have several implications for future research and support strategies in postgraduate medical training. Longitudinal studies are warranted to clarify how neurodevelopmental traits, psychological flexibility processes, burnout, depression/anxiety, and empathy influence one another over time. Future studies should also examine protective factors that may buffer residents from burnout and psychological distress, including work-related conditions and social support (9). Additionally, given that progress toward values was involved in the associations between ALTs and personal accomplishment, depression/anxiety, and empathy in the present study, future research could also examine whether a sense of meaning, intrinsic reward, and professional value derived from helping others functions as a related protective factor. This possibility is consistent with qualitative findings from emotionally demanding healthcare settings, in which care providers described meaning, joy, and professional value in their work despite exposure to highly distressing clinical situations (70). Future intervention studies should examine the specific process-based approaches—including those informed by ACT—that are most useful for residents with different neurodevelopmental trait patterns—for example, by targeting value-based action, cognitive defusion, or experiential acceptance according to individual needs (71). Finally, support strategies should avoid pathologizing neurodevelopmental traits and instead focus on creating training environments that recognize heterogeneity in both vulnerability and adaptive functioning. Such approaches may contribute to more individualized, inclusive, and preventive support for early-career physicians.

### 4.7 Limitations

This study has several limitations. First, its cross-sectional design precludes causal inference regarding the relationships between ALTs or ADHLTs and outcomes such as burnout and depression and anxiety symptoms. Accordingly, the indirect associations observed in the exploratory statistical mediation analyses should be interpreted as hypothesis-generating findings rather than evidence of temporal or causal mediation. Although ALTs and ADHLTs are neurodevelopmental traits that would generally be expected to precede psychological distress, the direction of these associations cannot be confirmed in the present design. Second, the study relied on self-report questionnaires, which may have introduced information bias. Although the instruments used have demonstrated validity, they are not equivalent to clinical diagnostic assessments. Third, not all eligible junior residents completed the survey, raising the possibility of selection bias. Additionally, no background information was collected regarding participants’ neurodevelopmental or mental health conditions, such as formal diagnoses of autism, ADHD, anxiety disorders, or depression, or regarding current psychiatric or pharmacological treatment. Therefore, residual confounding by active psychiatric conditions or ongoing treatment cannot be excluded, particularly for outcomes such as HADS scores and burnout dimensions. Recruitment bias, whereby individuals with neurodevelopmental or mental health conditions may have been more likely to participate, also cannot be excluded. Nevertheless, because approximately 80% of invited residents responded, the magnitude of this bias may have been limited. Fourth, the study did not assess all six core processes of psychological flexibility. Although the hexaflex model distinguishes six related but separable flexibility processes, prior work also suggests substantial shared variance among these processes, and studies may therefore focus on theoretically relevant components depending on their aims (72, 73). In the present study, we selected processes considered most relevant to burnout and psychological distress; however, future research would benefit from more comprehensive assessment of the full model. Fifth, the study was conducted at only two institutions. Although these hospitals may broadly reflect Japanese postgraduate training settings, broader inclusion of diverse training sites is needed to improve generalizability. Sixth, although the observed pattern of lower emotional exhaustion and depression and anxiety symptoms alongside higher depersonalization and lower personal accomplishment may reflect post-COVID-19 changes, the cross-sectional design limits firm conclusions. Longitudinal studies are needed to track changes in burnout and psychological distress over time. Nonetheless, a substantial proportion of participants in the current study also took part in a previous survey conducted among medical students between 2019 and 2022 (5), providing limited opportunities for historical comparison. Finally, these findings should not be interpreted as pathologizing neurodevelopmental traits, which exist along a continuum. Rather, the present results are intended to contribute to understanding variability in both vulnerability and adaptive functioning across individuals and to inform more nuanced and supportive approaches to physician well-being.

## 5 Conclusions

This study examined associations of ALTs and ADHLTs with multiple dimensions of psychological functioning among Japanese junior residents, including burnout, depression and anxiety symptoms, and physician–patient empathy. ALTs were associated with lower personal accomplishment, higher depression and anxiety symptoms, and lower empathy. ADHLTs were associated with higher emotional exhaustion.

The exploratory statistical mediation findings suggest that progress toward values may be particularly relevant to the associations of ALTs with personal accomplishment, depressive and anxiety symptoms, and empathy, whereas cognitive fusion may be particularly relevant to the association between ADHLTs and emotional exhaustion. These findings support future examinations of ACT-informed approaches targeting value-based action and cognitive defusion in residents with elevated neurodevelopmental traits.

## Data Availability

The raw data supporting the conclusions of this article will be made available by the authors, without undue reservation.

## Supplementary material

Supplementary Tables 1–3 Present simple slopes analyses of the association between AQ-J-21 and each outcome at low (-1 SD), mean, and high (+1 SD) levels of the ASRS Screener. **Supplementary Tables 1 and 2** report logistic regression results for binary outcomes, including ORs and 95% CIs, and **Supplementary Table 3** reports linear regression results for the continuous outcome of empathy (JSPE), including unstandardized coefficients and 95% CIs.

Supplementary Tables 4, 5 Present sensitivity analyses of the combined effects of ALTs and ADHLTs. In both tables, participants were categorized into 4 groups based on ALT and ADHLT status: high ALT/high ADHLT, low ALT/high ADHLT, high ALT/low ADHLT, and low ALT/low ADHLT (reference group). **Supplementary Table 4** presents logistic regression results for the binary outcomes of low personal accomplishment and high depression/anxiety, whereas **Supplementary Table 5** presents linear regression results for the continuous outcome of empathy (JSPE).

Supplementary Table 6 Presents sensitivity analyses using continuous MBI-EE, MBI-DP, and MBI-PA scores as outcomes. **Supplementary Table 7** presents sensitivity analyses using continuous HADS-Total, HADS-Anxiety, and HADS-Depression scores as outcomes. **Supplementary Table 8** presents detailed results of the age- and sex-adjusted exploratory statistical mediation models, including model fit indices; standardized path coefficients; and total, direct, and indirect effects.
