# Supplementary material for: Autistic- and attention-deficit/hyperactivity disorder-like traits: differential associations with burnout, depression and anxiety, and empathy among Japanese junior residents
Source: Front Psychiatry. 2026 Jul 9;17:1858994. doi: 10.3389/fpsyt.2026.1858994 (PMC13391826; doi:10.3389/fpsyt.2026.1858994)
Supplement: Supplementary Tables 1–3 — Present simple slopes analyses of the association between AQ-J-21 and each outcome at low (-1 SD), mean, and high (+1 SD) levels of the ASRS Screener. Supplementary Tables 1 and 2 report logistic regression results for binary outcomes, including ORs and 95% CIs, and Supplementary Table 3 reports linear regression results for the continuous outcome of empathy (JSPE), including unstandardized coefficients and 95% CIs. [file DataSheet1.pdf]

Supplementary Material

Supplementary Table S1. Simple slopes for the association between the AQ-J-21 score and low personal accomplishment (MBI-PA ≤ 33) at low (−1 SD), mean, and high (+1 SD) levels of the ASRS Screener score (logistic regression)

ASRS Screener level	Est (logit)	SE	z	p	OR	95% CI for OR
**−1 SD**	**0.44**	**0.13**	**3.45**	**0.0006**	**1.55**	**1.21–1.98**
**Mean**	**0.24**	**0.08**	**2.93**	**0.0034**	**1.27**	**1.08–1.49**
+1 SD	0.04	0.08	0.48	0.6300	1.04	0.88–1.23

Note. Est = estimate (log-odds coefficient from logistic regression); SE = standard error; z = Wald test statistic = Est / SE; p = two-tailed significance level; OR = odds ratio = exp(Est); CI = confidence interval (95% CI for OR = exp(Est ± 1.96 × SE)); ASRS Screener = Adult ADHD Self-Report Scale Screener. For values in bold, statistical significance set at p <0.05.

Supplementary Table S2. Simple slopes for the association between the AQ-J-21 score and high depression/anxiety (HADS-Total ≥ 20) at low (−1 SD), mean, and high (+1 SD) levels of the ASRS Screener score (logistic regression)

ASRS Screener level	Est (logit)	SE	z	p	OR	95% CI for OR
**−1 SD**	**0.34**	**0.13**	**2.75**	**0.0060**	**1.41**	**1.10–1.80**
**Mean**	**0.20**	**0.07**	**2.71**	**0.0068**	**1.22**	**1.06–1.41**
+1 SD	0.06	0.07	0.75	0.4560	1.06	0.91–1.22

Note. Est = estimate (log-odds coefficient from logistic regression); SE = standard error; z = Wald test statistic = Est / SE; p = two-tailed significance level; OR = odds ratio = exp(Est); CI = confidence interval (95% CI for OR = exp(Est ± 1.96 × SE)); ASRS Screener = Adult ADHD Self-Report Scale Screener. For values in bold, statistical significance set at p <0.05.

Supplementary Table S3. Simple slopes for the association between AQ-J-21 and JSPE scores at low (−1 SD), mean, and high (+1 SD) levels of the ASRS Screener score (linear regression)

ASRS Screener level	B	SE	t	p	95% CI for B
**−1 SD**	**−2.342**	**0.516**	**−4.54**	**0.000**	**−3.354 to −1.331**
**Mean**	**−1.564**	**0.341**	**−4.59**	**0.000**	**−2.232 to −0.896**
+1 SD	−0.786	0.434	−1.81	0.070	−1.637 to 0.065

Note. B = unstandardized regression coefficient from linear regression; SE = standard error; t = t-statistic = B / SE; p = two-tailed significance level; CI = confidence interval (95% CI for B = B ± 1.96 × SE); ASRS Screener = Adult ADHD Self-Report Scale Screener. For values in bold,

statistical significance set at p <0.05.

Supplementary Table S4. Sensitivity analysis of binary outcomes by ALT/ADHLT groups

**Outcome**	**Group**	**N**	**Cases**	**Adjusted OR**	**95% CI**	***p*-value**
Low personal accomplishment	High ALT + High ADHLT	13	10	1.05	[0.26 - 4.22]	0.947
	Low ALT + High ADHLT	22	20	3.33	[0.70 - 15.8]	0.13
	High ALT + Low ADHLT	22	20	3.47	[0.73 - 16.5]	0.117
	Low ALT + Low ADHLT (Ref)	91	70	1.00	—	—
High depression/anxiety	**High ALT + High ADHLT**	**13**	**4**	**4.85**	**[1.19 - 19.8]**	**0.028**
	**Low ALT + High ADHLT**	**22**	**7**	**5.29**	**[1.58 - 17.72]**	**0.007**
	**High ALT + Low ADHLT**	**22**	**8**	**6.88**	**[2.06 - 23.01]**	**0.002**
	Low ALT + Low ADHLT (Ref)	91	8	1.00	—	—

Note. ALT = autistic-like trait; ADHLT = ADHD-like trait; OR = odds ratio; CI = confidence interval; Ref = reference group. For values in bold, statistical significance set at p <0.05.

Supplementary Table S5. Sensitivity analysis of continuous outcomes by ALT/ADHLT groups

**Outcome**	**Group**	**B**	**95% CI**	**SE**	**t**	***p*-value**
JSPE (empathy)	**High ALT + High ADHLT**	**-8.49**	**[-16.75 - -0.22]**	**4.18**	**-2.03**	**0.044**
	Low ALT + High ADHLT	-1.85	[-8.59 - 4.90]	3.41	-0.54	0.590
	**High ALT + Low ADHLT**	**-13.36**	**[-20.14 - -6.58]**	**3.43**	**-3.89**	**0.000**
	Low ALT + Low ADHLT (Ref)	0.00	—	—	—	—

Note. ALT = autistic-like trait; ADHLT = ADHD-like trait; JSPE = Jefferson Scale of Physician Empathy; B = regression coefficient; CI = confidence interval; SE = standard error; t = t-statistic; Ref = reference group. For values in bold, statistical significance set at p <0.05.

Supplementary Table S6. Sensitivity analyses using continuous MBI subscale scores as outcomes

		**95% CI**				
**Variables**	**B**	**Lower**	**Upper**	**t**	***p*-value**	**Multiple R²**
MBI-EE						
Constant	-5.739	-21.838	10.360	-0.705	0.482	
Age	0.531	-0.060	1.123	1.775	0.078	
Sex (male)	-2.980	-5.960	0.000	-1.977	0.050	
AQ-J-21	0.224	-0.209	0.656	1.023	0.308	
**ASRS Screener**	**1.396**	**0.409**	**2.382**	**2.797**	**0.006**	
AQ-J-21 × ASRS Screener	0.224	-0.056	0.503	1.582	0.116	0.128
MBI-DP						
**Constant**	**20.549**	**9.167**	**31.932**	**3.569**	**<0.001**	
AQ-J-21	0.134	-0.172	0.439	0.863	0.389	
ASRS Screener	0.064	-0.633	0.761	0.182	0.856	
**Age**	**-0.462**	**-0.881**	**-0.044**	**-2.185**	**0.031**	
Sex (male)	0.634	-1.473	2.740	0.595	0.553	
AQ-J-21 × ASRS Screener	-0.082	-0.280	0.116	-0.821	0.413	0.048
MBI-PA						
**Constant**	**30.210**	**10.703**	**49.716**	**3.061**	**0.003**	
Age	-0.402	-1.119	0.314	-1.110	0.269	
Sex (male)	1.457	-2.153	5.068	0.798	0.426	
**AQ-J-21**	**-0.895**	**-1.419**	**-0.371**	**-3.378**	**0.001**	
ASRS Screener	0.562	-0.633	1.758	0.930	0.354	
AQ-J-21 × ASRS Screener	0.334	-0.005	0.673	1.950	0.053	0.091

Note. CI = confidence interval; MBI-EE = Maslach Burnout Inventory emotional exhaustion subscale; MBI-DP = Maslach Burnout Inventory depersonalization subscale; MBI-PA = Maslach Burnout Inventory personal accomplishment subscale; AQ-J-21 = 21-item Japanese version of the Autism-Spectrum Quotient; ASRS Screener = Adult ADHD Self-Report Scale Screener. AQ-J-21 and ASRS Screener scores were mean-centered before creating the interaction term. Multiple R^2 values were 0.128 for MBI-EE, 0.048 for MBI-DP, and 0.091 for MBI-PA. Adjusted R^2 values were 0.098, 0.014, and 0.059, respectively. VIFs ranged from 1.021 to 1.105 across all models. HC3 robust standard errors yielded the same overall conclusion: the ASRS Screener remained significantly associated with MBI-EE, the AQ-J-21 remained significantly associated with MBI-

PA, and the interaction terms were not significant. For values in bold, statistical significance set at *p* <0.05.

Supplementary Table S7. Sensitivity analyses using continuous HADS-Total, HADS-Anxiety, and HADS-Depression scores as outcomes

		**95% CI**				
**Variables**	**B**	**Lower**	**Upper**	**t**	***p*-value**	**Multiple R^2**
**HADS-Total**						
Constant	9.751	-0.629	20.132	1.857	0.065	
Age	0.155	-0.226	0.537	0.804	0.423	
Sex (male)	-0.783	-2.704	1.138	-0.805	0.422	
**AQ-J-21**	**0.653**	**0.375**	**0.932**	**4.633**	**<0.001**	
**ASRS Screener**	**0.894**	**0.258**	**1.530**	**2.778**	**0.006**	
AQ-J-21 × ASRS Screener	-0.149	-0.329	0.031	-1.636	0.104	0.200
**HADS-Anxiety**						
**Constant**	**8.778**	**2.172**	**15.383**	**2.627**	**0.010**	
Age	-0.044	-0.287	0.199	-0.356	0.722	
Sex (male)	-0.900	-2.123	0.322	-1.456	0.148	
**AQ-J-21**	**0.382**	**0.205**	**0.560**	**4.262**	**<0.001**	
**ASRS Screener**	**0.417**	**0.012**	**0.821**	**2.035**	**0.044**	
AQ-J-21 × ASRS Screener	-0.067	-0.182	0.048	-1.156	0.249	0.156
**HADS-Depression**						
Constant	0.974	-4.541	6.488	0.349	0.728	
Age	0.199	-0.004	0.402	1.940	0.054	
Sex (male)	0.118	-0.903	1.138	0.228	0.820	
**AQ-J-21**	**0.271**	**0.123**	**0.419**	**3.615**	**<0.001**	
**ASRS Screener**	**0.477**	**0.139**	**0.815**	**2.791**	**0.006**	
AQ-J-21 × ASRS Screener	-0.082	-0.178	0.014	-1.695	0.092	0.180

Note. CI = confidence interval; HADS-Total = Hospital Anxiety and Depression Scale total score; HADS-Anxiety = Hospital Anxiety and Depression Scale anxiety subscale; HADS-Depression = Hospital Anxiety and Depression Scale depression subscale; AQ-J-21 = 21-item Japanese version of the Autism-Spectrum Quotient; ASRS Screener = Adult ADHD Self-Report Scale Screener. AQ-J-21 and ASRS Screener scores were mean-centered before creating the interaction term.

Multiple R^2 values were 0.200 for HADS-Total, 0.156 for HADS-Anxiety, and 0.180 for HADS-Depression. Adjusted R^2 values were 0.172, 0.126, and 0.152, respectively. VIFs ranged from 1.021 to 1.105 across all models. HC3 robust standard errors yielded the same overall conclusion: AQ-J-21 remained significantly associated with all three outcomes, ASRS Screener remained significantly associated with HADS-Total and HADS-Depression and was borderline significant for HADS-Anxiety, and the interaction terms were not significant. For values in bold, statistical significance set at $p < 0.05$.

S8a. Model fit and explained variance

**Model**	**Indirect model**	**χ^2**	**df**	**CFI**	**TLI**	**RMSEA**	**SRMR**	**R^2 for outcome**
A	AQ-J-21 → VQ-P/WAAQ → MBI-PA	0.000	0	1.000	1.000	0.000	0.000	0.194
B	ASRS Screener → CFQ-7 → MBI-EE	0.000	0	1.000	1.000	0.000	0.000	0.170
C	AQ-J-21 → CFQ-7/VQ-P/WAAQ → HADS-T	0.000	0	1.000	1.000	0.000	0.000	0.521
D	AQ-J-21 → VQ-P → JSPE	0.000	0	1.000	1.000	0.000	0.000	0.198

S8b. Main path coefficients

**Model**	**Path**	**B**	**95% BCBOOT CI for B**	***p*-value**	**β (STDYX)**	**95% BCBOOT CI for β**
A	AQ-J-21 →VQ-P	-0.601	-0.884 to -0.293	<0.001	-0.330	-0.475 to -0.163
	VQ-P → MBI-PA	0.655	0.399 to 0.931	<0.001	0.378	0.243 to 0.517
	AQ-J-21 → WAAQ	-0.366	-0.666 to -0.025	0.023	-0.206	-0.359 to -0.007

	WAAQ → MBI-PA	0.060	-0.232 to 0.346	0.686	0.034	-0.129 to 0.196
	AQ-J-21 → MBI-PA	-0.340	-0.849 to 0.224	0.216	-0.108	-0.273 to 0.074
B	ASRS Screener → CFQ-7	1.665	0.697 to 2.699	0.001	0.280	0.119 to 0.437
	CFQ-7 → MBI-EE	0.281	0.106 to 0.466	0.002	0.271	0.099 to 0.428
	ASRS Screener → MBI-EE	1.105	0.146 to 2.122	0.027	0.179	0.024 to 0.329
C	AQ-J-21 → CFQ-7	0.566	0.102 to 1.007	0.014	0.221	0.035 to 0.385
	CFQ-7 → HADS-T	0.296	0.209 to 0.390	<0.001	0.424	0.307 to 0.544
	AQ-J-21 → VQ-P	-0.601	-0.884 to -0.293	<0.001	-0.330	-0.475 to -0.163
	VQ-P → HADS-T	-0.312	-0.461 to -0.170	<0.001	-0.317	-0.457 to -0.175
	AQ-J-21 → WAAQ	-0.366	-0.666 to -0.025	0.023	-0.206	-0.359 to -0.007

	WAAQ → HADS-T	-0.173	-0.295 to -0.027	0.010	-0.172	-0.307 to -0.026
	AQ-J-21 → HADS-T	0.263	0.032 to 0.538	0.045	0.147	0.019 to 0.305
D	AQ-J-21 → VQ-P	-0.601	-0.884 to -0.293	<0.001	-0.330	-0.475 to -0.163
	VQ-P → JSPE	0.684	0.329 to 1.074	<0.001	0.300	0.150 to 0.449
	AQ-J-21 → JSPE	-1.020	-1.850 to -0.301	0.008	-0.246	-0.428 to -0.075

S8c. Total, direct, and indirect effects

**Model**	**Effect**	**B**	**SE**	***p*-value**	**95% BCBOOT CI for B**	**β (STDYX)**	**95% BCBOOT CI for β**
A	Total effect	-0.755	0.270	0.005	-1.282 to -0.211	-0.240	-0.396 to -0.066
	Direct effect	-0.340	0.275	0.216	-0.849 to 0.224	-0.108	-0.273 to 0.074
	Total indirect effect	-0.416	0.151	0.006	-0.759 to -0.161	-0.132	-0.232 to -0.054
	Specific indirect via VQ-P	-0.394	0.134	0.003	-0.685 to -0.163	-0.125	-0.214 to -0.054
	Specific indirect via WAAQ	-0.022	0.061	0.720	-0.166 to 0.089	-0.007	-0.053 to 0.028
B	Total effect	1.573	0.528	0.003	0.557 to 2.607	0.255	0.096 to 0.397
	Direct effect	1.105	0.500	0.027	0.146 to 2.122	0.179	0.024 to 0.329

	Total indirect effect	0.468	0.224	0.037	0.151 to 1.057	0.076	0.025 to 0.160
	Specific indirect via CFQ-7	0.468	0.224	0.037	0.151 to 1.057	0.076	0.025 to 0.160
C	Total effect	0.681	0.144	<0.001	0.401 to 0.957	0.381	0.215 to 0.522
	Direct effect	0.263	0.132	0.045	0.032 to 0.538	0.147	0.019 to 0.305
	Total indirect effect	0.418	0.132	0.002	0.164 to 0.680	0.234	0.095 to 0.370
	Specific indirect via CFQ-7	0.167	0.078	0.032	0.033 to 0.338	0.094	0.017 to 0.184
	Specific indirect via VQ-P	0.187	0.069	0.007	0.074 to 0.366	0.105	0.044 to 0.195
	Specific indirect via WAAQ	0.063	0.044	0.147	0.003 to 0.179	0.035	0.002 to 0.100
D	Total effect	-1.432	0.353	<0.001	-2.152 to -0.762	-0.344	-0.497 to -0.190
	Direct effect	-1.020	0.388	0.008	-1.850 to -0.301	-0.246	-0.428 to -0.075
	Total indirect effect	-0.411	0.168	0.014	-0.813 to -0.143	-0.099	-0.190 to -0.035
	Specific indirect via VQ-P	-0.411	0.168	0.014	-0.813 to -0.143	-0.099	-0.190 to -0.035

Note. All models were adjusted for age and sex by regressing the process variables and outcome variables on these covariates. Residual covariances among process variables were allowed when multiple process variables were included. Because these models were saturated or just-identified with zero degrees of freedom, the fit indices were reported descriptively and were not interpreted as evidence of model adequacy. χ^2 = chi-square test statistic; df = degrees of freedom; CFI =

comparative fit index; TLI = Tucker–Lewis index; RMSEA = root mean square error of approximation; SRMR = standardized root mean square residual; R^2 = coefficient of determination; B = unstandardized coefficient; β = standardized coefficient (STDYX); CI = confidence interval; BCBOOT = bias-corrected bootstrap; AQ-J-21 = 21-item Japanese version of the Autism-Spectrum Quotient; ASRS Screener = Adult ADHD Self-Report Scale Screener; CFQ-7 = Cognitive Fusion Questionnaire-7; VQ-P = Valuing Questionnaire Progress subscale; WAAQ = Work-related Acceptance and Action Questionnaire; MBI-PA = Maslach Burnout Inventory personal accomplishment subscale; MBI-EE = Maslach Burnout Inventory emotional exhaustion subscale; HADS-T = Hospital Anxiety and Depression Scale total score; JSPE = Jefferson Scale of Physician Empathy.
